# Standardization of Clinical Assessment and Sample Collection Across All PERCH Study Sites

**DOI:** 10.1093/cid/cix077

**Published:** 2017-05-27

**Authors:** Jane Crawley, Christine Prosperi, Henry C. Baggett, W. Abdullah Brooks, Maria Deloria Knoll, Laura L. Hammitt, Stephen R. C. Howie, Karen L. Kotloff, Orin S. Levine, Shabir A. Madhi, David R. Murdoch, Katherine L. O’Brien, Donald M. Thea, Juliet O. Awori, Charatdao Bunthi, Andrea N. DeLuca, Amanda J. Driscoll, Bernard E. Ebruke, Doli Goswami, Melissa M. Hidgon, Ruth A. Karron, Sidi Kazungu, Nana Kourouma, Grant Mackenzie, David P. Moore, Azwifari Mudau, Magdalene Mwale, Kamrun Nahar, Daniel E. Park, Barameht Piralam, Phil Seidenberg, Mamadou Sylla, Daniel R. Feikin, J. Anthony G. Scott, for the PERCH Study Group, Katherine L. O’Brien, Orin S. Levine, Maria Deloria Knoll, Daniel R. Feikin, Andrea N. DeLuca, Amanda J. Driscoll, Nicholas Fancourt, Wei Fu, Laura L. Hammitt, Melissa M. Higdon, E. Wangeci Kagucia, Ruth A. Karron, Mengying Li, Daniel E. Park, Christine Prosperi, Zhenke Wu, Scott L. Zeger, Nora L. Watson, Jane Crawley, David R. Murdoch, W. Abdullah Brooks, Hubert P. Endtz, Khalequ Zaman, Doli Goswami, Lokman Hossain, Yasmin Jahan, Hasan Ashraf, Stephen R. C. Howie, Bernard E. Ebruke, Martin Antonio, Jessica McLellan, Eunice Machuka, Arifin Shamsul, Syed M.A. Zaman, Grant Mackenzie, J. Anthony G. Scott, Juliet O. Awori, Susan C. Morpeth, Alice Kamau, Sidi Kazungu, Karen L. Kotloff, Milagritos D. Tapia, Samba O. Sow, Mamadou Sylla, Boubou Tamboura, Uma Onwuchekwa, Nana Kourouma, Aliou Toure, Shabir A. Madhi, David P. Moore, Peter V. Adrian, Vicky L. Baillie, Locadiah Kuwanda, Azwifarwi Mudau, Michelle J. Groome, Henry C. Baggett, Somsak Thamthitiwat, Susan A. Maloney, Charatdao Bunthi, Julia Rhodes, Pongpun Sawatwong, Pasakorn Akarasewi, Donald M. Thea, Lawrence Mwananyanda, James Chipeta, Phil Seidenberg, James Mwansa, Somwe wa Somwe, Geoffrey Kwenda

**Affiliations:** 1 Centre for Tropical Medicine and Global Health, Nuffield Department of Clinical Medicine, University of Oxford, United Kingdom;; 2 Department of International Health, International Vaccine Access Center, Johns Hopkins Bloomberg School of Public Health, Baltimore, Maryland;; 3 Global Disease Detection Center, Thailand Ministry of Public Health–US Centers for Disease Control and Prevention Collaboration, Nonthaburi;; 4 Division of Global Health Protection, Center for Global Health, Centers for Disease Control and Prevention, Atlanta, Georgia;; 5 International Centre for Diarrhoeal Disease Research, Bangladesh (icddr,b), Dhaka and Matlab;; 6 Department of International Health, Johns Hopkins Bloomberg School of Public Health, Baltimore, Maryland;; 7 Kenya Medical Research Institute–Wellcome Trust Research Programme, Kilifi;; 8 Medical Research Council Unit, Basse, The Gambia;; 9 Department of Pediatrics, University of Auckland, and; 10 Centre for International Health, University of Otago, Dunedin, New Zealand;; 11 Division of Infectious Disease and Tropical Pediatrics, Department of Pediatrics, Center for Vaccine Development, Institute of Global Health, University of Maryland School of Medicine, Baltimore, and; 12 Bill & Melinda Gates Foundation, Seattle, Washington;; 13 Medical Research Council, Respiratory and Meningeal Pathogens Research Unit, and; 14 Department of Science and Technology/National Research Foundation, Vaccine Preventable Diseases Unit, University of the Witwatersrand, Johannesburg, South Africa;; 15 Department of Pathology, University of Otago, and; 16 Microbiology Unit, Canterbury Health Laboratories, Christchurch, New Zealand;; 17 Center for Global Health and Development, Boston University School of Public Health, Massachusetts, and Departments of; 18 Epidemiology and; 19 International Health, Center for Immunization Research, Johns Hopkins Bloomberg School of Public Health, Baltimore, Maryland;; 20 Centre pour le Développement des Vaccins (CVD-Mali), Bamako;; 21 Murdoch Childrens Research Institute, Melbourne, Australia;; 22 London School of Hygiene & Tropical Medicine,United Kingdom;; 23 Department of Paediatrics and Child Health, Chris Hani Baragwanath Academic Hospital and University of the Witwatersrand, South Africa;; 24 University Teaching Hospital, Lusaka, Zambia;; 25 Milken Institute School of Public Health, Department of Epidemiology and Biostatistics, George Washington University, Washington, District of Columbia;; 26 Nakhon Phanom Provincial Health Office, Nakhon Phanom, Thailand;; 27 Department of Emergency Medicine, University of New Mexico, Albuquerque, and; 28 Division of Viral Diseases, National Center for Immunizations and Respiratory Diseases, Centers for Disease Control and Prevention, Atlanta, Georgia; and; 29 Department of Infectious Disease Epidemiology, London School of Hygiene & Tropical Medicine, United Kingdom; 30 Johns Hopkins Bloomberg School of Public Health, Baltimore, Maryland; 31 Bill & Melinda Gates Foundation, Seattle, Washington; 32 Centers for Disease Control and Prevention [CDC], Atlanta, Georgia; 33 The Emmes Corporation, Rockville, Maryland; 34 Nuffield Department of Clinical Medicine, University of Oxford, United Kingdom; 35 University of Otago, Christchurch, New Zealand; 36 ICDDR,b, Dhaka and Matlab, Bangladesh; 37 Medical Research Council, Basse, The Gambia; 38 KEMRI–Wellcome Trust Research Programme, Kilifi, Kenya; 39 Division of Infectious Disease and Tropical Pediatrics, Department of Pediatrics, Center for Vaccine Development, Institute of Global Health, University of Maryland School of Medicine, Baltimore, Maryland and Centre pour le Développement des Vaccins (CVD-Mali), Bamako, Mali; 40 Respiratory and Meningeal Pathogens Research Unit, University of the Witwatersrand, Johannesburg, South Africa; 41 Thailand Ministry of Public Health—US CDC Collaboration, Nonthaburi, Thailand; 42 Boston University School of Public Health, Boston, Massachusetts and University Teaching Hospital, Lusaka, Zambia

**Keywords:** pneumonia, childhood, hospital, training, standardization.

## Abstract

**Background.:**

Variable adherence to standardized case definitions, clinical procedures, specimen collection techniques, and laboratory methods has complicated the interpretation of previous multicenter pneumonia etiology studies. To circumvent these problems, a program of clinical standardization was embedded in the Pneumonia Etiology Research for Child Health (PERCH) study.

**Methods.:**

Between March 2011 and August 2013, standardized training on the PERCH case definition, clinical procedures, and collection of laboratory specimens was delivered to 331 clinical staff at 9 study sites in 7 countries (The Gambia, Kenya, Mali, South Africa, Zambia, Thailand, and Bangladesh), through 32 on-site courses and a training website. Staff competency was assessed throughout 24 months of enrollment with multiple-choice question (MCQ) examinations, a video quiz, and checklist evaluations of practical skills.

**Results.:**

MCQ evaluation was confined to 158 clinical staff members who enrolled PERCH cases and controls, with scores obtained for >86% of eligible staff at each time-point. Median scores after baseline training were ≥80%, and improved by 10 percentage points with refresher training, with no significant intersite differences. Percentage agreement with the clinical trainer on the presence or absence of clinical signs on video clips was high (≥89%), with interobserver concordance being substantial to high (AC1 statistic, 0.62–0.82) for 5 of 6 signs assessed. Staff attained median scores of >90% in checklist evaluations of practical skills.

**Conclusions.:**

Satisfactory clinical standardization was achieved within and across all PERCH sites, providing reassurance that any etiological or clinical differences observed across the study sites are true differences, and not attributable to differences in application of the clinical case definition, interpretation of clinical signs, or in techniques used for clinical measurements or specimen collection.

Current pneumonia treatment and prevention strategies are based mainly on data obtained from large clinical studies carried out in the 1980s. One such study, sponsored by the Board of Science and Technology for International Development (BOSTID), National Academy of Sciences, yielded valuable information on the pathogens present during acute respiratory infections (ARIs) in children <5 years old from resource-limited countries [1]. However, interpretation of the wide range of reported ARI incidence rates was complicated in part by the lack of a standardized case definition at the 10 participating study sites [2]. A subsequent literature review of pneumonia etiology studies, conducted between 2000 and 2010 on children aged <5 years, revealed wide disparity in case definitions, specimen collection techniques, and laboratory methods, which increased the complexity of data collation and analysis [3]. Other studies have demonstrated substantial interclinician variation in the interpretation of clinical signs of severe disease in children and young infants [4–7]. Standardization of the clinical [8], radiological [9], laboratory [10], and data management methods [11] at all PERCH sites has been prioritized since inception, as we wished to ensure that any observed variation in pneumonia etiology between sites was not attributable to methodological differences. The objectives of the clinical standardization program were to ensure that study staff (1) adhered strictly to the clinical case definitions; (2) were consistent in their assessment, recognition, and interpretation of clinical signs; (3) used standardized equipment and techniques for obtaining clinical measurements; and (4) used standardized methods for obtaining key clinical samples for laboratory testing. This paper describes the PERCH clinical standardization program of clinical training, retraining, and staff assessment that ran throughout the study.

## METHODS

### Study Sites

At all sites (Table 1), clinical assessment and enrollment of PERCH cases and controls were carried out by doctors, nurses, and clinical officers (health workers with at least 3 years of formal clinical training). Nurses and field workers or research assistants took anthropometric measurements, assisted clinical staff with procedures, and identified and located PERCH community controls.

**Table 1.** Profile of Pneumonia Etiology Research for Child Health (PERCH) Study Sites

Country	Training Language	Study Site	Setting	Start of Enrollment	Staff Responsible for Enrollment of PERCH Cases and/or Controls		
					Cadre	No. (%)	Total
Kenya	English	Kilifi	Rural	August 2011	Doctor	3 (13)	23
					CO^a^	18 (78)	
					Nurse	2 (9)	
South Africa	English	Johannesburg	Urban	August 2011	Doctor	1 (8)	13
					Nurse	12 (92)	
Zambia	English	Lusaka	Urban	October 2011	Doctor	4 (23)	17
					CO^a^	3 (18)	
					Nurse	10 (59)	
The Gambia	English	Basse	Rural	November 2011	Doctor	7 (23)	31
					Nurse	24 (77)	
Mali	French^c^	Bamako	Urban	January 2012	Doctor	12 (67)	18
					Nurse	6 (33)	
Bangladesh	Bangla^c^ & English	Dhaka	Urban	January 2012	Doctor^b^	37 (100)	37
		Matlab	Rural	January 2012			
Thailand	Thai^c^ & English	Sa Kaeo	Mixed	January 2012	Doctor Nurse	2 (11) 17 (89)	19
		Nakhon Phanom	Mixed	February 2012			
						Total	158

Abbreviations: CO, clinical officer; PERCH, Pneumonia Etiology Research for Child Health.

^a^Clinical officers are health workers with at least 3 years of formal clinical training.

^b^In Bangladesh, all enrollment decisions were made by doctors, although nurses helped to identify potential cases and controls.

^c^Multiple-choice questions (MCQs) were translated into French (Mali) or Thai (Thailand); staff in Bangladesh took MCQs in English.

### Preparatory Phase

The PERCH case definition (Table 2) was based on the 2005 World Health Organization (WHO) clinical definition of severe and very severe pneumonia [8]. The definition relies on the presence of prespecified clinical signs, without information from chest radiograph (CXR) or pulse oximetry. The PERCH enrollment period predated the 2013 reclassification of severe and very severe pneumonia by the WHO [12].

**Table 2.** Pneumonia Etiology Research for Child Health (PERCH) Clinical Case Definition of Severe and Very Severe Pneumonia^a^

Case	Sign or Symptom	Detailed Definition
Pneumonia (nonsevere)	Cough or difficulty breathing plus fast breathing	
	Cough	On history and/or examination
	Difficulty breathing	Fast, labored, deep, irregular, or noisy breathing
	Fast breathing	Respiratory rate (breaths/min): ≥60 (<2 mo); ≥50 (2–11 mo); ≥40 (1–5 y)
Severe pneumonia	Cough or difficulty breathing plus lower chest wall indrawing	
	Lower chest wall indrawing	Inward movement of the lower bony chest wall on inspiration; child must be calm and not crying
Very severe pneumonia	Cough or difficulty breathing plus any of the following signs or symptoms^b^:	
	Central cyanosis	Blue discoloration of lips, gums, and tongue; should be assessed under good lighting conditions
	Head nodding	Flexion of the head with inspiration; more commonly seen in young children and infants. Most easily seen if child is upright
	Unable to drink or breastfeed	This must be observed in the clinical environment, by study staff: <2 mo: feeding poorly (eg, poor attachment to breast, weak suck) ≥2 mo: inability to take anything (fluids or solids) by mouth
	Vomiting everything	This must be observed in the clinical environment, by study staff: Child is given a drink: if child has not vomited by the end of the clinical assessment, and before study procedures are carried out, then s/he is not “vomiting everything”
	Lethargy or unconsciousness	AVPU score^c^ = V, P, or U
	Convulsions this illness	Based on detailed description by parent or guardian. For inclusion in PERCH, convulsions must be prolonged (≥15 min) or multiple (≥2 within a 24-h period during the current illness)^d^

Abbreviation: PERCH, Pneumonia Etiology Research for Child Health.

^**a**^Based on World Health Organization (2005) clinical case definition of severe and very severe pneumonia (Pocket Book of Hospital Care for Children).

^b^Lower chest wall indrawing is not a defining sign of very severe pneumonia as it may disappear if the child becomes exhausted.

^c^AVPU score: (1) clinician first assesses whether the child is alert; A = alert (child takes an age-appropriate interest in their environment); if child not alert, clinician tests, in sequence, V, P, and U, stopping when the child gives a positive response; (2) clinician calls the child’s name without simultaneously touching him or her; V = response to voice (any consistent visual, verbal, or motor response to voice); (3) clinician presses on the base of the child’s fingernail using a pencil or pen; P = response to pain (child withdraws digit); (4) U = unresponsive or unconscious (no response to pain).

^d^Definition of complex febrile seizure used by American Academy of Pediatrics (Pediatrics 2011; 127: 389–94); PERCH adopted a stringent definition of “convulsions this illness” to avoid enrolling large numbers of children with cough and simple febrile seizures.

Through a series of teleconferences and 2 face-to-face meetings between all PERCH principal investigators (PIs), consensus was achieved on how to elicit, recognize, and interpret each of the signs and symptoms comprising the PERCH clinical case definition (Table 2), and on the choice of methods and equipment for obtaining key clinical measurements (pulse oximetry, anthropometry, respiratory rate) and clinical samples (nasopharyngeal [NP] and oropharyngeal [OP] swabs, induced sputum [IS], lung aspirates, blood, urine).

Training materials and advice were sought from a wide variety of sources (see Acknowledgments). Many of the clinical video clips, audio recordings and photographs were recorded at PERCH sites by the principal trainer (J. C.), with written informed consent from the patient’s parents or guardians.

### Training Courses

Initial clinical standardization training occurred at all sites immediately prior to a period of pilot enrollment. All sites enrolled to the main study for 24 months, with refresher training carried out in the first and second year.

The initial 3-day training and subsequent 2-day refresher training courses were conducted at all sites by the principal trainer, with support from site project leaders. All cadres of PERCH staff (doctors, nurses, clinical officers, research assistants, and field workers) were trained together; interested local non-PERCH clinicians were invited to participate.

Training courses comprised lectures, discussion of case scenarios in small groups, practical sessions, and ward-based clinical teaching. The initial training lectures covered the background to the PERCH study, rationale for clinical standardization, recognition of the critically ill child, clinical assessment of the child with cough or difficulty breathing, vital signs, pulse oximetry, techniques for collection of NP/OP swabs, and anthropometry. Discussion of PERCH case scenarios, designed to test the trainees’ ability to identify signs and symptoms that constitute study inclusion and exclusion criteria, took place in groups of 8–10 people, each group being led by the principal trainer and/or a local facilitator. Trainees were divided into groups of 5–8 for hands-on instruction in clinical assessment. Staff members were asked to conduct clinical assessments on children, and were assessed on their ability to elicit and correctly interpret clinical signs.

Practical skills were taught through training videos, demonstrations, and hands-on practice in small groups, with key points highlighted in summary lectures. Clinically stable children acted as subjects for the anthropometry training. Staff learned NP/OP swab collection by practicing on each other. Clinicians from sites where IS samples were routinely collected from children (Kenya, The Gambia, South Africa) trained staff from the other 4 sites. The clinical team in Kenya reviewed video recordings of the collection procedures in The Gambia and South Africa, to ensure that they were consistent with procedures in Kenya. IS training was included in the refresher courses, as was guidance on reducing blood culture contamination rates through improved phlebotomy technique. Ethical approval to perform diagnostic percutaneous needle lung aspiration among PERCH cases was only obtained in The Gambia, Mali, South Africa, and Bangladesh. Clinicians from The Gambia (where lung aspiration is performed frequently on children with focal consolidation on CXR [13]) trained PERCH staff from the other 3 countries. Pleural aspirates and gastric aspirates were not included in the training as they were not designated PERCH procedures, but were carried out as routine hospital procedures if clinically indicated.

All courses finished with a multiple-choice question (MCQ) examination, presentation of certificates, prizes for those achieving top scores, and a group photograph. All participants were invited to provide feedback, using a Likert scale to grade the quality of different course components.

### Clinical Standardization Guidelines

Guidelines summarizing key information from the training program were distributed to all staff at the time of refresher training, with an electronic version made available on the internal PERCH study website.

### Training Website

A training website (www.perchtraining.org), developed in association with a company specializing in digital healthcare (see Acknowledgments), had the following objectives: (1) to act as a repository for the clinical standardization training materials, thereby supporting the training of any new staff who had missed their initial site training course; (2) to provide continuing training of all PERCH staff throughout study enrollment; and (3) to facilitate regular evaluation of all PERCH clinical staff, and comparison of staff performance within and across sites.

The website contained all lectures from the initial training course, which could be streamed or downloaded as lectures with recorded voice-over, or as PowerPoint presentations. When internet speeds were slow, staff accessed training materials from DVDs, which had been distributed to all sites at the start of the study. At several sites, limited access to personal computers meant that project leaders downloaded the MCQs and organized the evaluations as classroom sessions. The website baseline training was supplemented by on-site training in practical skills and ward-based clinical teaching, both coordinated by the local PERCH study leader. On completion of the online course, trainees were required to take the same MCQ examination as those who had participated in face-to-face training. Trainees achieving a score of 80% or more were able to download a certificate from the website. The website also contained 2 additional MCQ examinations and a video quiz (see Evaluation).

### Evaluation

MCQ examinations were conducted after initial baseline training, immediately before and after each refresher course, and online after 10 months and 20 months of enrollment. An online video quiz was used to assess interobserver variation in interpretation of clinical signs at 20 months. Checklist evaluation of practical skills was performed at the end of the first year.

MCQs were designed to test knowledge and understanding of the screening, consent and enrollment process, and the recognition and correct interpretation of key clinical signs, particularly those included in the WHO definitions of severe and very severe pneumonia. Each of the 10–20 MCQs contained a typical PERCH case scenario, plus, in most cases, a photograph or short video of a clinical sign. Answers to each question were provided at the end of the quiz, once all of the questions had been answered, with explanatory notes highlighting key learning points. Staff scoring <80% in the MCQ administered after baseline training were required to repeat selected lectures and the quiz, while staff scoring <80% after refresher training received additional training from their site-specific trainer.

The video quiz assessed the ability of clinical staff to identify 6 clinical signs (lower chest wall indrawing [LCWI], head nodding, deep breathing, central cyanosis, nasal flaring, alert child). Clinical staff were shown 35 video clips (10 videos of LCWI, the defining clinical feature of WHO severe pneumonia, and 5 videos of each of the other clinical signs). Each video lasted approximately 10 seconds, and clinicians had to decide whether a specific clinical sign was present or not.

Local clinical standardization trainers observed PERCH nurses and field workers carrying out anthropometry, IS, and NP/OP swab collection. Scored checklists (Supplementary Tables 1–3) were used to award points for key predefined procedural steps, the resulting percentage score providing a measure of procedural competence.

### Statistical Analysis

Median percentage scores and interquartile range (IQR) were calculated for MCQ tests and checklists. Median MCQ scores before and after refresher training were compared using the Wilcoxon signed-rank test. The distribution of results across participants was compared within and across study sites. Results were stratified by professional cadre and by whether staff assessed both cases and controls, or controls only. Differences between groups were examined with the Kruskal-Wallis test.

For each of the 6 clinical signs in the video quiz (35 videos in total), individual responses were used to assess the percentage agreement between the clinical staff and the principal trainer, who was the designated “gold standard.” Calculation of Fleiss’ κ and the Gwet AC1 statistic, which is less affected by low prevalence than the κ statistic, were used to measure the degree of interobserver variability [14–16].

Kaplan-Meier curves were constructed to illustrate the proportion of PERCH clinical staff remaining in the study, from the time of baseline clinical standardization training. Curves were censored when staff members left the study, or on completion of PERCH enrollment.

## RESULTS

### Training Courses

Between March 2011 and August 2013, a total of 32 training courses were conducted at 8 study sites in 7 countries. Of 331 staff attending 1 or more courses, 45 (14%) were interested local clinical staff, not directly involved in the study. Feedback from course participants was positive, with 90% of all course components being graded as “very good” (4/5) or “excellent” (5/5).

Initial (baseline) clinical standardization training took place over a 6-month period between March and September 2011. At each site, training occurred immediately prior to a period of pilot enrollment, and a median of 5 months (range, 4–9 months) before the start of the study. Seventy staff members joined PERCH after the initial training course at their site, and received baseline training from their site project leader and/or the training website. In South Africa, baseline training was repeated 6 months after the start of enrollment, due to extensive staff turnover during the pilot period.

The first round of refresher training took place a median of 7 (range, 5–11) months and the second round a median of 18 months (range, 14–21) after the start of the study. A national nurses strike in Kenya during 2012 delayed refresher training by 3 months. At all other sites, training and enrollment continued uninterrupted, despite extensive flooding in Thailand during 2011, civil war in Mali during 2012–2013, and political instability in Bangladesh during 2013.

### Evaluation

**Figure 1.**
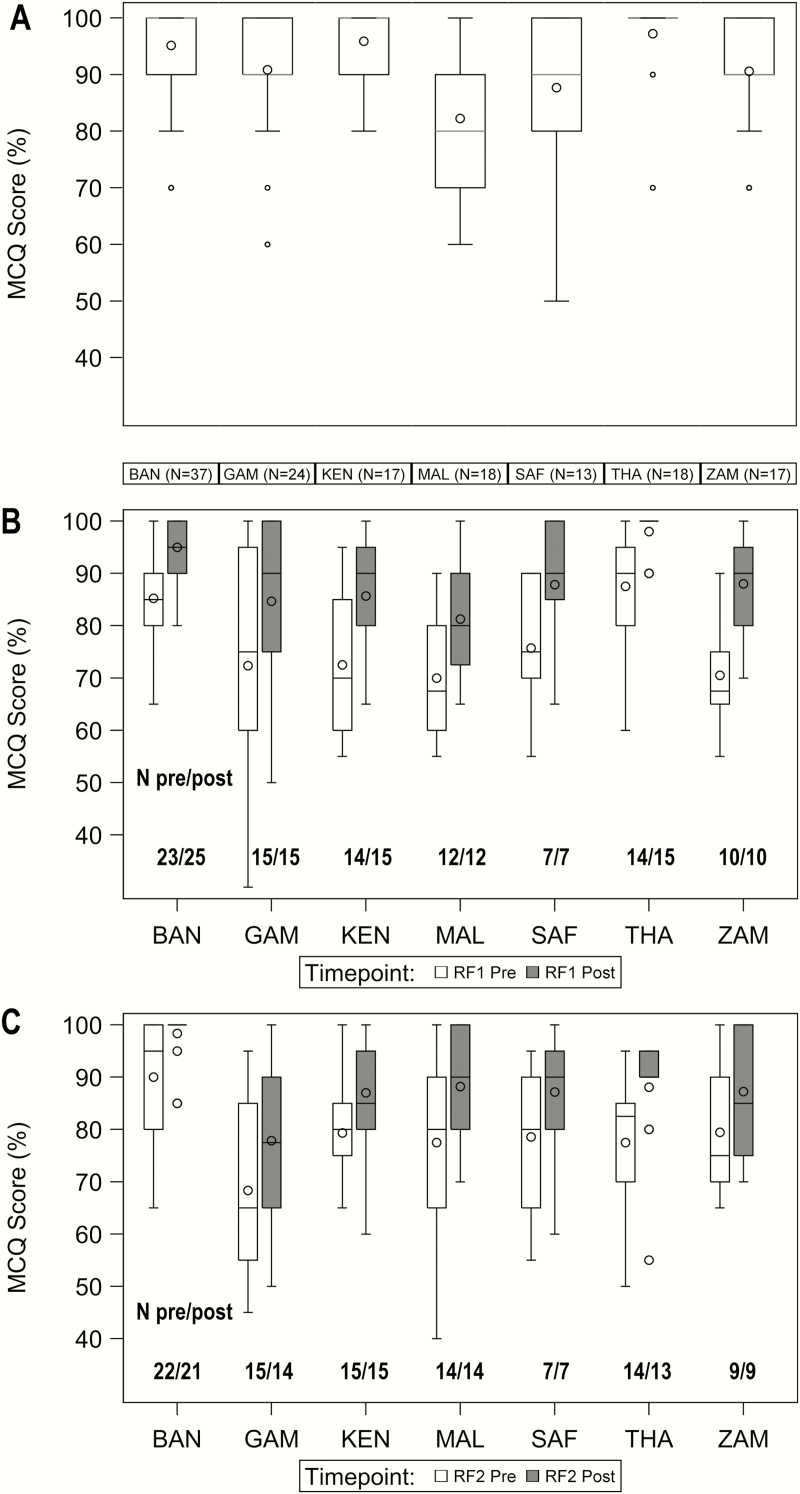
Distribution of multiple choice question (MCQ) scores by site and training time-point: postbaseline training (*A*), pre- and postrefresher training 1 (*B*), and refresher training 2 (*C*). Boxplots display the distribution of MCQ scores. The number beneath each boxplot indicates the number of Pneumonia Etiology Research for Child Health (PERCH) clinicians and nurses who took the MCQ at each site. The diamond and horizontal line within the boxes represent the mean and median, respectively. The box reflects the interquartile range (IQR) and the whiskers extend to 1.5 multiplied by the IQR in either direction, or maximum and minimum values (if no outliers). The circle indicates outliers (values lying outside 1.5 multiplied by the IQR). Abbreviations: BAN, Bangladesh; GAM, The Gambia; KEN, Kenya; MAL, Mali; MCQ, multiple-choice question; pre, pre-course MCQ; post, post-course MCQ; RF1, refresher training 1; RF2, refresher training 2; SAF, South Africa; THA, Thailand; ZAM, Zambia.

MCQ and video quiz results are presented for the 158 doctors, clinical officers, and nurses who enrolled PERCH cases and/or controls. High rates of staff turnover meant that only 57 of 158 (36.1%) of those who received baseline training completed all 7 MCQs plus the video quiz, but at each evaluation time-point MCQ scores were available for a median of 94% (IQR, 87%–100%) of the eligible staff at all sites (Table 3). Median scores were ≥80% at each point of testing, and improved with refresher training by a median of 10 percentage points. There was significant heterogeneity (*P* < .001) in the range of baseline training scores between sites, with South Africa and Mali having the greatest range of scores and Thailand the least variability (Figure 1A). Refresher training scores are shown in Figure 1B and 1*C*. The proportion of staff attaining a score of ≥80% rose from 54.7% and 60.4% before refresher training 1 and 2, respectively, to 84.9% and 82.8% after training (Table 3). The difference between pre- and postcourse scores (excluding those attaining 100% in the precourse MCQ) did not vary significantly (*P* > .8) between sites. Median precourse MCQ scores were significantly lower among nurses and clinical officers compared to doctors (Table 4); nurses who assessed controls only scored lower than those who assessed both cases and controls, though (with the exception of prerefresher training 1) this failed to reach statistical significance.

**Table 3.** Multiple-Choice Question Scores for Clinical Staff Assessing Pneumonia Etiology Research for Child Health (PERCH) Cases and/or Controls, by Evaluation Time-Point (All Study Sites)

Evaluation (MCQ) Time-Point	No. of Clinical Staff^a^	No. of Staff With MCQ Score^b^	MCQ Score		Improvement With Refresher Training^c^	
			Median % Score (IQR)	Percentage Scoring ≥80	Median Difference (Post- Pre) (IQR)	Percentage With Improved Scores
Postbaseline training^d^	158	144	100 (90–100)	87.5		
Prerefresher training 1	110	95	80 (65–90)	54.7	10 (10–20)e	93.1
Postrefresher training 1	110	99	90 (85–100)	84.9		
Online MCQ 1	110	103	90 (80–100)f	90.3		
Prerefresher training 2	105	96	80 (70–90)	60.4	10 (5–15)e	88.5
Postrefresher training 2	105	93	90 (80–100)	82.8		
Online MCQ 2	110	99	90 (75–100)f	74.8		

Abbreviations: IQR, interquartile range; MCQ, multiple-choice question; PERCH, Pneumonia Etiology Research for Child Health.

^a^The reduction in staff numbers after baseline training reflects staff loss, which was greatest during the pilot period and early months of recruitment.

^b^Missing values: (*i*) Baseline training (n = 14): All 14 staff received baseline training; 9 joined PERCH during the last 6 months of recruitment and trained online, but failed to take the final MCQ; 2 were site trainers, 1 of whom had translated all of the MCQ questions, answers, and explanations into Thai; 3 MCQ scores were mislaid. (*ii*) Refresher training (median, 11 [range, 7–15]): staff absent from refresher training 1 or 2 or the online MCQs were on sick, compassionate, annual, or maternity leave, or were carrying out essential ward duties.

^c^Excludes staff scoring 100% on the prerefresher training.

^d^Baseline training refers to the training that all staff underwent at the time of joining the study; it does not relate to a specific time-point, as new staff members were recruited throughout the study.

e*P* < .001 with Wilcoxon signed-rank test.

f*P* = .17 with Kruskal-Wallis test (no significant difference in distribution of scores between online MCQ1 and MCQ2).

**Table 4.** Multiple-Choice Question Scores by Cadre and Role

Evaluation Time-Point	MCQ % Score, Median (IQR)							MCQ % Score, Median (IQR)				
	Doctor		Clinical Officer		Nurse			Nurses Sees Cases and Controls		Nurses Sees Controls Only		
	No.		No.		No.		*P* Value^a^	No.		No.		*P* Value^a^
Postbaseline training	64	100 (90–100)	16	100 (90–100)	64	90 (80–100)	.07	49	90 (90–100)	15	90 (70–100)	.10
Prerefresher training 1	42	85 (75–90)	13	75 (60–85)	40	75 (62.5–85)	.02	31	80 (65–95)	9	65 (55–65)	.02
Postrefresher training 1	44	95 (90–100)	13	90 (80–95)	42	90 (75–100)	.05	32	90 (80–100)	10	77.5 (65–90)	.08
Online MCQ 1	46	100 (90–100)	17	90 (80–90)	40	90 (80–100)	.03	30	90 (80–100)	10	90 (80–100)	.91
Prerefresher training 2	39	90 (80–100)	15	85 (75–85)	42	70 (60–85)	<.001	31	75 (65–85)	11	65 (55–75)	.13
Postrefresher training 2	36	100 (95–100)	13	85 (85–95)	44	82.5 (72.5–95)	<.001	31	90 (75–95)	13	80 (70–90)	.46
Online MCQ 2	42	100 (95–100)	14	65 (60–80)	43	85 (65–95)	<.001	33	85 (60–95)	10	87.5 (80–95)	.59

Abbreviations: IQR, interquartile range; MCQ, multiple-choice question; PERCH, Pneumonia Etiology Research for Child Health.

^a^ *P* value obtained by Kruskal-Wallis test.

Checklist evaluations of practical skills were carried out on 105 of 166 (63%) staff performing NP/OP swabs, 64 of 112 (57%) staff collecting IS samples, and 107 of 166 (64%) staff conducting anthropometry. Analyzing all sites combined, median checklist scores were 92% (IQR, 90%–96) for NP/OP swabs, 96% (IQR, 90%–98) for IS, and 95% (IQR, 88%–100) for anthropometry, with a median score of >82% for each of the 3 skills when analyzing by site.

The video quiz took place during the final 4 months of enrollment at each site. Ninety-six of 110 current staff members participated, of whom 42 (44%) were nurses, 40 (42%) doctors, and 14 (14%) clinical officers. Percentage agreement between participants and the clinical trainer was high (≥89%) for all clinical signs (Table 5). Interobserver concordance was moderate for central cyanosis (AC1 statistic, 0.54); substantial for LCWI, deep breathing, nasal flaring, and the alert child (AC1, 0.62–0.82); and excellent for head nodding (AC1, 0.88).

**Table 5.** Agreement With Principal Trainer and Interobserver Agreement for Select Clinical Signs

	Agreement With Trainer^a^		Interobserver Agreement	
Clinical Sign	No. of Videos	Percentage Agreement With Trainer, Median (IQR)	AC1^b^	κ^b^
LCWI	10	89.1 (85.4–95.8)	0.62	0.62
Head nodding	5	99.0 (95.8–99.0)	0.88	0.87
Deep breathing	5	92.7 (92.7–99.0)	0.82	0.80
Central cyanosis	5	90.2 (75.8–94.6)	0.54	0.54
Nasal flaring	5	95.8 (93.8–99.0)	0.79	0.68
Alert child	5	94.8 (83.3–97.9)	0.62	0.62

Abbreviations: IQR, interquartile range; LCWI, lower chest wall indrawing.

^a^One hundred ten staff members who assessed Pneumonia Etiology Research for Child Health (PERCH) cases and/controls were available for the video quiz. Ninety-six (87%) staff participated in the video quiz (14 missing values).

^b^For both the AC1 and κ statistic, a value of 0 indicates no agreement beyond chance, while a value of 1 denotes perfect agreement. Values of ≤0.40 are generally indicative of poor agreement, 0.41–0.60 moderate agreement, 0.61–0.80 substantial agreement, and >0.80 excellent agreement.

### Staff Retention

**Figure 2.**
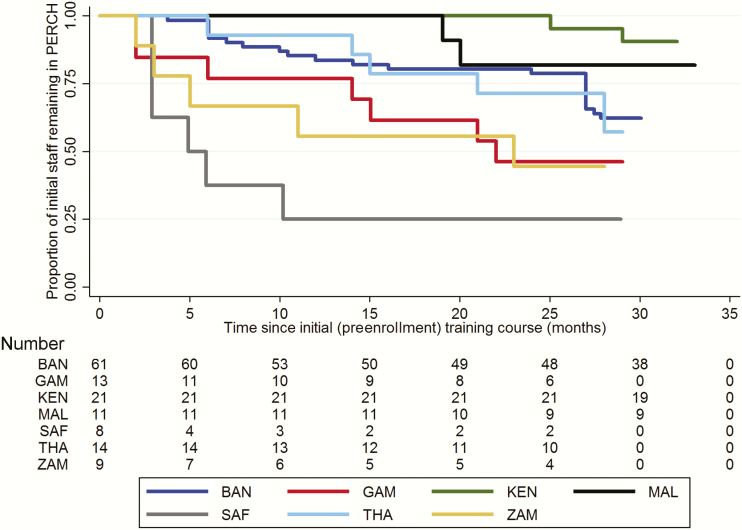
Staff retention during the course of the Pneumonia Etiology Research for Child Health (PERCH) study, by site. Kaplan-Meier graph displaying the proportion of staff attending the initial baseline training (N = 137) who remained with the study; analysis includes all staff members regardless of whether or not they enrolled study participants. Drops represent staff members leaving the study over time. The table beneath the graph indicates the number of staff members who remained in the study over time. Abbreviations: BAN, Bangladesh; GAM, The Gambia; KEN, Kenya; MAL, Mali; SAF, South Africa; THA, Thailand; ZAM, Zambia.

Figure 2 provides an intersite comparison of staff retention for 137 staff members who attended the initial training course at their site, prior to the start of study enrollment. Retention varied by site (log-rank test for equality of survivor function across sites: *P* < .001) and cadre, with retention of clinical officers (86% [18/21] of whom were at the Kenya site) being significantly higher than retention of nurses and doctors (log-rank test, *P* = .016). Retention over 24 months of enrollment was high (>80%) in Kenya and Mali; moderate (40%–70%) in Bangladesh, Thailand, Zambia, and The Gambia; and low (<30%) in South Africa.

## DISCUSSION

Although staff training is an important component of all clinical trials, most studies fail to document its content or evaluate and report on its effectiveness [17]. By means of MCQs, a video quiz, and checklist evaluation of practical skills, we assessed key knowledge and clinical skills of PERCH staff throughout the duration of the study. Despite considerable challenges posed by staff turnover, language differences, intersite variation in the number and cadre of staff performing clinical assessments, and political and geographic factors beyond our control, a satisfactory level of clinical standardization was achieved within and across all study sites. Because of clinical standardization, we consider that the variable proportion of very severe pneumonia cases at different PERCH sites, from 10% in Bangladesh, where screening took place in an outpatient clinic, to approximately 50% among hospitalized children in Mali and Kenya, is a true reflection of intersite differences in case severity.

MCQs were administered at the end of baseline training and at regular intervals throughout the study. To answer questions correctly, staff needed thorough knowledge of the PERCH case definition and inclusion and exclusion criteria, and the ability to recognize and interpret key clinical signs from the accompanying video clips. The lower MCQ scores attained in Mali following baseline training may have been because the 3-day course concluded 1 day early due to extenuating circumstances, and was delivered in French by a nonnative speaker. In Thailand and Bangladesh, courses were delivered in both English and Thai or Bangla, and MCQ scores at these sites were comparable to the scores from countries where English is more widely spoken. At all sites and time points, doctors attained significantly higher MCQ scores than clinical officers and nurses, who generally spend a shorter period of time in professional clinical training. The nurses who only assessed healthy controls scored worse than nurses assessing both cases and controls, probably because they were exposed to fewer children with clinical signs.

Clinical video has been shown previously to be an effective way of testing agreement between clinicians on the presence or absence of clinical signs [6], despite the obvious difference from the “real-life” clinical situation, in which a clinician’s judgement is affected by information other than an isolated clinical sign. The same study showed that health workers of different cadres and varying levels of clinical experience could correctly identify clinical signs from video recordings for which there was high proportionate agreement between experts [6]. Clinical signs are not, however, always clear-cut in real-life. To this end, the PERCH video quiz included a random selection (approximately 20%) of “gray” cases—namely, those in which a clinical sign (eg, LCWI) was present but subtle, making it genuinely difficult to decide on its presence or absence. Despite this, percentage agreement between staff and the trainer was ≥89% for all 6 clinical signs in the quiz, while interobserver agreement (agreement between participants) varied from “moderate” for central cyanosis, a clinical sign which is easily missed in African children and which is difficult to photograph or film successfully, to “substantial” or “excellent” for the other clinical signs. Good-quality clinical video clips are a valuable and scarce resource, and we hope that the video clips available on the PERCH clinical standardization training website (www.perchtraining.org) will be useful for other clinical researchers.

Although the PERCH clinical standardization program successfully attained its objectives, a number of useful lessons have been learned. It would have been informative to evaluate staff knowledge and skills prior to the initial training course, as this would have provided a useful baseline comparator for the subsequent MCQ scores. The improvement in MCQ scores with refresher training suggests that it would have been valuable to have had more regular refresher training courses at each site, coordinated by local site trainers. Limited availability of personal computers and slow internet speeds reduced the utility of the training website at several of the study sites. These shortcomings are not shared by mobile phone technology, which could provide a useful alternative platform for training and evaluation. It took time to obtain a sufficient number of good-quality video clips of relevant clinical signs, and consequently the video quiz took place during the final 4 months of enrollment, by which time many of the original PERCH staff had left the study. It would have been preferable to organize the quiz at the start of enrollment, and repeat it during the second year. Although the checklist evaluations of practical skills were useful training and evaluation tools, they were time-consuming and were consequently performed on approximately 60% of the relevant study staff. High rates of staff turnover emphasized the importance of establishing a robust system for training new staff outside of the regular training schedule. Turnover was lowest among clinical officers, which may reflect their longer-term clinical attachments.

There is increasing recognition that public health policy should be based on data that are globally representative. Enhanced connectivity, the widespread availability of powerful computing, statistical and data management tools, and the advent of funders willing to pay for large networked studies have increased the feasibility of conducting large, multicountry research studies. Ensuring that the clinical and laboratory data obtained during the course of such studies are robust, standardized, and comparable is of paramount importance. The results of the PERCH clinical standardization program give us confidence that any etiological or clinical differences observed across the study sites are true differences, and not attributable to differences in application of the clinical case definition or differences in techniques used for clinical measurements or specimen collection. We hope that the methods, results, and lessons learned from the PERCH clinical standardization program will usefully inform other researchers embarking on large-scale clinical or epidemiological studies of pneumonia or other major causes of childhood morbidity and mortality.

## Supplementary Data

Supplementary materials are available at *Clinical Infectious Diseases* online. Consisting of data provided by the authors to benefit the reader, the posted materials are not copyedited and are the sole responsibility of the authors, so questions or comments should be addressed to the corresponding author.

## Supplementary Material

Supplementary_Material

## Notes

***Author contributions.*** J. C. designed the clinical standardization materials and was the principal trainer. O. S. L., K. L. O., D. R. F., D. R. M., M. D. K., L. L. H., H. C. B., W. A. B., S. R. C. H., K. K. L., S. A. M., J. A. G. S., D. M. T., S. C. M., and R. A. K. conceived and designed the study and supervised study conduct. C. P., A. N. D., M. D. K., J. A. G. S., L. L. H., and D. R. F. participated in the analysis and interpretation of results and drafting of the manuscript. J. O. A., C. B., A. N. D., A. J. D., B. E. E., D. G., M. M. H., R. A. K., S. K., N. K., G. M., D. P. M., A. M., M. M., K. N., D. E. P., B. P., P. S., and M. S. were involved in the study conduct, data collection, and/or data management. All authors review and approved the manuscript. J. C. had final responsibility for the decision to submit for publication.

***Acknowledgments.*** We thank the following individuals and organizations for providing clinical video clips and training materials: WHO, Department of Child and Adolescent Health and Department of Nutrition; Professor Mike English; Professor Kathryn Maitland; Professor Karen Kotloff; GlaxoSmithKline; Rale Repository; COPAN Diagnostics. We are grateful to Dr David Peel and Dr Irwin Shorr for their valuable advice on pulse oximetry and anthropometry, respectively. We thank Incuna Ltd for their invaluable support in the development of the PERCH clinical standardization website. We offer sincere thanks to the patients and families who participated in this study.

We are grateful to the following members of the original study teams for running the studies and collecting data:

***Financial support.*** PERCH was supported by the Bill & Melinda Gates Foundation (grant number 48968 to the International Vaccine Access Center, Department of International Health, Johns Hopkins Bloomberg School of Public Health). J. A. G. S. was supported by a clinical fellowship from The Wellcome Trust of Great Britain (award number 098532).

***Supplement sponsorship.*** This article appears as part of the supplement “Pneumonia Etiology Research for Child Health (PERCH): Foundational Basis for the Primary Etiology Results,” sponsored by a grant from the Bill & Melinda Gates Foundation to the PERCH study of Johns Hopkins Bloomberg School of Public Health, Baltimore, Maryland.

***Potential conflicts of interest.*** M. D. K. has received funding for consultancies from Merck, Pfizer, and Novartis, and grant funding from Merck. L. L. H. has received grant funding from Pfizer and GlaxoSmithKline. K. L. K. has received grant funding from Merck Sharp & Dohme. S. A. M. has received honoraria for advisory board membership from the Bill & Melinda Gates Foundation, Pfizer, Medimmune, and Novartis; has received institutional grants from GSK, Novartis, Pfizer, Minervax, and the Bill & Melinda Gates Foundation; and has served on speaker’s bureaus for Sanofi Pasteur and GSK. K. L. O. has received grant funding from GSK and Pfizer and participates on technical advisory boards for Merck, Sanofi Pasteur, PATH, Affinivax, and ClearPath. All other authors report no potential conflicts. All authors have submitted the ICMJE Form for Disclosure of Potential Conflicts of Interest. Conflicts that the editors consider relevant to the content of the manuscript have been disclosed.
