# Supplementary material for: Standardization of Clinical Assessment and Sample Collection Across All PERCH Study Sites
Source: Clin Infect Dis. 2017 May 27;64(Suppl 3):S228–37. doi: 10.1093/cid/cix077 (PMC5447838; doi:10.1093/cid/cix077)
Supplement: Supplementary_Material [file cix077_suppl_Supplementary_Material.pdf]

Supplementary Table 1. PERCH Anthropometry Checklist

Staff name: **Date of assessment:** **Assessor initials:**

**Weight**	**Score** 0 = not done 1 = done	**Score**
Explain procedure to mother and child	0 or 1	
Remove child's shoes & minimize clothing (infants: dry nappies)	0 or 1	
Children ≥2 years: stand still on centre of scale<br>Children <2 years/infants or children who are unable to stand: weigh carer; weigh carer + child; difference = weight of child	0 or 1	
Measure weight to nearest 0.1kg<br>For scales weighing to 2 decimal points: if 0.05kg & above round up; if <0.05kg round down	0 or 1	
Say weight out loud & write it down immediately (check that order of digits is not inverted)	0 or 1	
Show weight to mother & write it in child's health card, if available	0 or 1	
**Total score (weight)**	**Total** (max = 6)	

**Height** **(children ≥2 years)**	**Length** **(children <2 years)**	**Score** 0 = not done 1 = done	**Score**
Explain procedure to mother and child	Explain procedure to mother and child	0 or 1	
Must have an assistant (can be the mother, if no other staff available)	Must have an assistant (can be the mother, if no other staff available)	0 or 1	
Remove shoes/socks; remove hair ornaments; compress braids	Remove shoes, socks & bulky nappies; remove hair ornaments; compress braids	0 or 1	
Child stands upright in centre of board; hands by side	Child lies supine on the board (must be straight & flat)	0 or 1	
Back of child's head, shoulders, buttocks, calves & heels must touch the backboard	Top of child's head must touch the headboard	0 or 1	
Child's must look forward; direction of gaze is at 90 degrees to vertical plane	Child must look directly upwards; direction of gaze is at 90 degrees to horizontal plane	0 or 1	
Compress abdomen for full height	Soles of both feet MUST be flat against the movable footboard & at 90 degrees to the horizontal plane	0 or 1	
Lower head-board onto top of child's head; read & call out height to nearest 0.1cm	Hold footboard against soles of feet; read & call out length to nearest 0.1cm	0 or 1	
Assistant should record height & show it to measurer for checking	Assistant should record length & show it to measurer for checking	0 or 1	
Show height to mother & write it in child's health card, if available	Show length to mother & write it in child's health card, if available	0 or 1	

**Total score (height or length)**	**Total** (max = 10)	

Anthropometry checklist (continued)

**Mid upper arm circumference (MUAC)**	**Score**	**Score**
Explain procedure to mother and child	0 = not done<br>1 = done	
Identify & fully expose the LEFT arm	0 = right arm<br>1 = left (correct) arm	
Locate tip of shoulder: raise child's left arm & identify groove of shoulder joint; place your index finger in groove & mark tip of shoulder	0 = wrong technique<br>1 = correct technique	
With child's left elbow at 90 degrees (palm up), measure distance from tip of shoulder to tip of elbow; if using MUAC tape, the 2 arrows on the tape should be aligned with the tip of the shoulder (=0cm); the mid-point (half the distance between the tip of shoulder & tip of elbow) is then marked on the child's arm	0 = wrong technique<br>1 = correct technique	
Measurement of MUAC: 1) child's arm hangs down straight; 2) MUAC tape is wrapped round arm directly over the mid-point, with tip of tape passing through slot on right side of window; 3) MUAC tape should rest against the skin (must not compress the tissues or be loose) & MUAC is read at the centre of the window between the 2 arrows; 4) MUAC measurement (to nearest 0.1cm) should be said out loud & written down immediately	Score from 0 to 4 (one point for each step)	
Show MUAC to mother & write it in child's health card, if available	0 = not done<br>1 = done	
**Total score (MUAC)**	**Total** (max = 9)	

**Anthropometry score**	
**Score (weight)**	
**Score (height or length)**	
**Score (MUAC)**	
**TOTAL anthropometry score**<br>(weight + height or length + MUAC scores)<br>(Max = 25)	
**TOTAL % anthropometry score**<br>(Total score x 4)	**%**

Supplementary Table 2. PERCH induced sputum (IS) checklist

Staff name: **Date of assessment:** **Assessor initials:**

Procedure	Score
**Ask clinician to list the 5 contraindications to IS:**<br><br>1) convulsion within past 24 hours; 2) inability to protect airway; 3) severe bronchospasm; 4) oxygen saturation <92% for >10 minutes while on supplemental oxygen; 5) clinician considers that child is too sick for IS.<br><br>Score: 0 to 5 (score one point for each contraindication)	
Child must be nil by mouth for at least 2 hours prior to the start of the IS procedure<br><br>Score: 0 = no; 1 = yes (Nil by mouth for ≥2 hours)	
**Advance preparation:** obtain N95 face mask; non-sterile gloves; salbutamol inhaler & spacer or salbutamol nebulizer solution; nebulizer and face mask; hypertonic saline; normal saline flush; container; oxygen source; connecting tubing; suction pump (CHECK it is working); nasogastric (NG) tubes (sizes 6 & 8Fr); sputum trap; pulse oximeter & sensors; respiratory rate timer; laboratory forms; CRF 07*; cool box.<br><br>Score: 0 = >2 items missing; 1 = 1 or 2 items missing; 2 = no items missing	
**Explain procedure to mother;** explanation should cover the following 5 points:<br><br>1) rationale for doing IS; 2) what will be involved; 3) child will cry but procedure is safe & will not harm the child; 4) mother/assistant should hold child firmly to reduce movement & minimise discomfort; 5) ask mother if she has any questions.<br><br>Score: 0 = no explanation; 1 = explanation includes 1-3 of the above points; 2 = explanation includes ≥4 of above points	
**Attach pulse oximeter** (Note**:** pulse oximeter must remain continuously attached to child throughout the IS procedure) & record the following 4 baseline observations:<br><br>1) oxygen saturation; 2) oxygen requirement; 3) respiratory rate; 4) AVPU score.<br><br>Score: from 0 to 4 (score one point for each observation)	
**Health & safety:**<br><br>1) procedure must be carried out in well-ventilated room; 2) put on N95 mask; 3) wash hands (must use soap or antiseptic solution); 4) put on gloves.<br><br>Score: from 0 to 4 (score one point for each of the above steps)	
**Position child in mother's/assistant's arms:**<br><br>Infant or small child: chest to chest; mother's/assistant's left arm round child's body & right hand on his head;<br>**OR**<br>Older child: sitting facing forward on mother's/assistant's lap; mother's/assistant's right arm round arms, and left hand on child's forehead; child's legs held between mother's/assistant's legs.<br><br>Score: 0 = inadequate positioning ; 1 = secure positioning;	

**Administer salbutamol:**<br><br>1) give 2 puffs (2 x 100 mcg) of salbutamol from metered dose inhaler into spacer; 2) WAIT for 10 seconds or 10 breaths between each puff, and for 5 minutes after second puff.<br>**OR**<br>1) administer 2.5mg salbutamol solution via nebulizer; 2) WAIT until nebulizer solution has completely disappeared from chamber (full dose given)<br><br>Score: from 0 to 2 (score one point for each step)	
**Administration of hypertonic saline:**<br><br>1) add 5mls 5% hypertonic saline to nebuliser chamber; 2) position mask correctly on child's face & turn on nebuliser; 3) nebulise for 10 minutes or until all hypertonic saline has disappeared; 4) connect sputum trap via tubing to suction pump.<br><br>Score: from 0 to 4 (score one point for each of the above steps)	
**Gently percuss the chest** (5-10 taps on upper and lower quadrants of right & left posterior chest wall) during administration of hypertonic saline, if appropriate.<br><br>Score: 0 = percussion not done; 1 = percussion performed or not indicated (child >2 years)	
**Measure distance to which nasogastric (NG) tube should be inserted:**<br><br>1) NG tubing should remain in packaging during measurement to avoid contamination or, if unpackaged, must not touch child's face; 2) measure distance from ear lobe to base of nostril.<br><br>Score: from 0 to 2 (score one point for each of the above steps)	
**Obtain specimen:**<br><br>1) wipe child's nose to remove secretions; 2) extend child's neck slightly; 3) start respiratory rate timer; 4) insert NG tube to full measured distance (must reach the posterior nasopharynx, where resistance should be felt); 5) instruct assistant to apply suction; 6) STOP suction & withdraw catheter when catheter is half filled with secretions or when a maximum of 30 seconds have elapsed (whichever occurs first).<br><br>Score: from 0 to 6 (score one point for each of the above steps)	
**Transfer specimen:**<br><br>1) place tip of NG tube in 5mls vial of normal saline and apply suction until all the saline has been aspirated; 2) disconnect tubing from specimen container and cap the container tightly; 3) fix pre-printed labels to specimen container; 4) record time that specimen was obtained on the label; 5) place container in cool box for transport to laboratory.<br><br>Score: from 0 to 5 (score one point for each step)	
Ask clinician to state the 4 times at which post-IS observations (oxygen requirement, oxygen saturation, respiratory rate, AVPU score) should be performed:<br><br>1) Immediately after IS; 2) 30 minutes after IS; 3) 2 hours after IS; 4) 4 hours after IS.<br><br>Score from 0 to 4 (score one point for each)	
Ask clinician to list the 4 events that are considered Serious Adverse Events (SAEs) if they occur within 4 hours of IS (score one point for each): 1) drop in oxygen saturation to <92% for 10 minutes or more, necessitating additional supplemental oxygen; 2) deterioration in AVPU score; 3) new requirement for bronchodilator or increased frequency of bronchodilator treatment; 4) Death<br><br>Score: from 0 to 4	

Ask clinician to list the 2 clinical indications for stopping IS, and the 1 criterion for subsequently restarting it: 1) Oxygen saturation drops to ≤88% for ≥60 seconds; 2) Oxygen saturation drops to 89-91% for ≥60 seconds despite supplemental oxygen; 3) IS can be restarted if child's clinical condition improves to baseline status<br><br>Score: from 0 to 3 (score one point for each)	
**TOTAL SCORE** (maximum = 50)	
**Percentage (%)** (Total score x 2)	**%**

* CRF 07 (case report form used to detail IS collection procedural steps and specimen collection for PERCH cases).

Supplementary Table 3. PERCH NP/OP swab checklist

Staff name: **Date of assessment:** **Assessor initials:**

Procedure	Score
**NP & OP swabs**	
Advance preparation: assemble non-sterile gloves, 3 swabs (1 flocked 2 rayon), media (*VTM & STGG), labels, tongue depressor, cool box.<br><br>Score: 1 = one or more items are missing; 2 = no items are missing	
**Explain procedure to mother;** this should include the following 5 points:<br><br>1) reason for taking NP/OP swabs; 2) what is involved; 3) explain that child will cry but procedure is entirely safe; 4) emphasise importance of holding child firmly to reduce movement & minimise discomfort; 5) ask mother if she has any questions.<br><br>Score: 0 = no explanation; 1 = explanation includes 1 to 3 of the above points; 2 = explanation includes 4 or more of the above points.	
**Position child in mother's/assistant's arms:**<br>Infant or small child: chest to chest, mother's/assistant's left arm round child's body & right hand on his head.<br>Older child: sitting facing forward on mother's/assistant's lap; mother's/assistant's right arm round child's arms & left hand on child's forehead; child's legs held between mother's/assistant's legs<br><br>Score: 0 = inadequate positioning ; 1 = secure positioning	
Wash hands & put on gloves<br><br>Score: 0 = one or none; 1 = both	
**NP swab**	
Measure insertion distance: 1) Flocked swab must remain in packaging during measurement; 2) measure distance from tragus (front) of ear to base of nostril (if child has nasal discharge this should be wiped away with a tissue)<br><br>Score: from 0 to 2 (score one point for each step)	
Insert NP swab: 1) Swab should be held between thumb & index finger; 2) Insert swab into medial side of nostril (alongside the nasal septum); 3) Swab should remain horizontal throughout insertion (i.e. parallel to the hard palate); 4) Insert swab to full measured length (NOT just 1-2cm)<br><br>Score: from 0 to 4 (score one point for each step)	
Take sample: When swab is fully inserted, rotate 180 degrees to right and left and remove immediately<br><br>Score: 0 (not done) or 1	
Transfer swabs to media: 1) First NP swab is placed in VTM; 2) Second NP swab in STGG (check lid closure); 3) Both samples must be labelled; 4) Samples are placed in cool box or fridge.<br><br>Score: from 0 to 4 (score one point for each step)	
**OP swab**	
MUST use tongue depressor (spatula) to visualise tonsils<br><br>Score: 0 (tongue depressor NOT used) or 1 (tongue depressor used)	
Take samples: 1) Touch both tonsil beds and posterior oro-pharynx with swab, rotating swab 180 degrees to right and left; 2) Place OP swab in same tube of VTM as first NP swab.<br><br>Score: from 0 to 2 (score one point for each step)	
**TOTAL SCORE** (maximum = 20)	

**% score** (total score x 5)	**%**

*VTM = viral transport medium; STGG = skim milk-tryptone-glucose-glycerin medium (bacterial transport medium).

Acknowledgements:

PERCH Expert Group. William C. Blackwelder, Harry Campbell, John A. Crump, Adegoke Falade, Menno D de Jong, Claudio Lanata, Kim Mulholland, Shamim Qazi, Cynthia G. Whitney.

Pneumonia Methods Working Group. Robert E Black, Zulfiqar A Bhutta, Harry Campbell, Thomas Cherian, Derrick W Crook, Menno D de Jong, Scott F Dowell, Stephen M Graham, Keith P Klugman, Claudio F Lanata, Shabir A Madhi, Paul Martin, James P Nataro, Franco M Piazza, Shamim A Qazi, and Heather J Zar.

PERCH Contributors:

Bangladesh: Kamrun Nahar, Arif Uddin Sikdir, Sharifa Yeasmin, Dilruba Ahmed, Muhammad Ziaur Rahman, Muhammad Yunus, Muhammad Al Fazl Khan, Muhammad Jubayer Chisti, Abu Sadat Muhammad Sayeem, Shahriar Bin Elahi, Mustafizur Rahman; **The Gambia:** Michel Dione, Emmanuel Olutunde, Peter Githua, Ogochukwu Ofordile, Rasheed Salaudeen, David Parker; **Kenya:** Shebe Mohamed, Siti Ndaa, Micah Silaba, Neema Muturi, Angela Karani, Sammy Nyongesa, Anne Bett, Daisy Mugo, Salim Mwarumba, Robert Musyimi, Andrew Brent, James Nokes, David Mulewa, Joyce Sande, John Odhiambo, Joshua Wambua, Nuru Kibirige, Caroline Mulunda, Hellen Mjalla, Norbert Katira, Karen Dama, Loice Masha, Christine Mutunga, Mwanajuma Ngama, Stephen Mangi, Riziki Anthony, Mwarua Yubu, Elijah Wakili, Benson Katana, Shoboi Mgunya, Emmanuel Mumba, Benedict Mver, George Kuria, Felix Githinji, Norbert Kihuha, Boniface Jibendi, Tahreni Bwanaali, Agustus Kea; **Mali:** Nana Kourouma, Aliou Toure, Mahamadou Diallo, Breana Barger-Kamate, Mariam Samake, Seydou Sissoko, Abdoul Aziz Maiga, Mariam Samake, Toumani Sidibe, Mariam Sylla, Aziz Diakite, Bassirou Diarra; **South Africa:** Azwidihwi Takalani, Andrea Hugo, Susan Nzenze, Ndulela Titi, Mmabatho Selela, Malebo Motiane, Minah Nkuna, Nonhlanhla Tsholetsane, Sibonsile Moya, Debra Katisi, Tondani Netshishivhe, Lerato Mapetla, Gudani Singo, Simphiwe Gasa, Cece Mgenge, Nozipho Mthunzi, Nombulelo Monedi, Tanja Adams, Shafeeka Mangera, Jeannette Wadula, Peter Tsaagane, Jenifer L. Vaughan, Sakina Loonat, Martin Hale, Sugeshnee Pather, Mariëtte Middel, Siobhan Trenor, Palesa Morailane, Ntombi Maya, Rene Sterley, Charné Combrinck, Given Malete, Lerato Qoza, Grizelda Liebenberg, Hendrik van Jaarsveld, Zunaid Kraft, Lisa-Marie Mollentze, Lourens Combrinck, Tsholofelo Mosome; **Thailand:** Sununta Henchaichon, Dr. Tussanee Amornintapichet, Dr. Somchai Chuananont, Toni Whistler, Juraiporn Ratanodom, Patranuch Sapchookul, Ornuma Sangwichian, Sirirat Makprasert, Manoon Hirunsalee, Possawat Jorakate, Anek Kaewpan, Duangkamol Siludjai, Apiwat Lapamnouysup, Dr. Wantana Paveenkittiporn, Waraporn Ubonphen, Dr. Peera Areerat, Ms.Yupapan Wannachaiwong, Ms Tewa Faipet, Ms Punnat Natnarakorn, Ms Ahchanan Sacharone, Mr.Winai Makmool, Ms. Kanlaya Sornwong, Ms. Promporn Sansuriwong, Ms. Ratchanida Potiya, Ms. Wasana Hongsawong, Ms.Wipa Matchaikhen, Ms.

Thatsanawan Chaiyabil, Ms.Piyapai Wannarach, Ms Chamaiporn Wadeesirisak, Mr. Yuttapong Norapet, Mattana Bangkung, Mr. Barameht Piralam, Sathapana Naorat, Anchalee Jatapai, Prasong Srisaengchai, Dr. Leonard Peruski, Ms.Dawan Phaensoongnoen, Ms.Tussaaorn Klangprapan, Ms.Narawadee Dumrongdee, Ms.Atchara Srithongkham, Mr. Piyawut Noinont, Ms. Pornthip Kamlee, Ms.Siyapa Mongkornsuk; **Zambia:** Justin Mulindwa, Musaku Mwenechanya, John Mwaba, Magdalene Mwale, Julie Duncan, Kazungu Siazele, Muntanga Mapeni, Emily Hammond;
